# Constitutive Expression of an Apple *FLC3-like* Gene Promotes Flowering in Transgenic Blueberry under Nonchilling Conditions

**DOI:** 10.3390/ijms20112775

**Published:** 2019-06-06

**Authors:** Xiaojuan Zong, Yugang Zhang, Aaron Walworth, Elise M. Tomaszewski, Pete Callow, Gan-yuan Zhong, Guo-qing Song

**Affiliations:** 1 Plant Biotechnology Resource and Outreach Center, Department of Horticulture, Michigan State University, East Lansing, MI 48824, USA; xjzong812@163.com (X.Z.); zyg4458@163.com (Y.Z.); walwort8@msu.edu (A.W.); tomasz39@msu.edu (E.M.T.); callow@msu.edu (P.C.); 2 Shandong Institute of Pomology, Shandong Academy of Agricultural Sciences, Taian 271000, China; 3 Qingdao Key Laboratory of Genetic Improvement and Breeding in Horticultural Plants, College of Horticulture, Qingdao Agricultural University, Qingdao 266109, China; 4 Grape Genetics Research Unit, USDA-ARS, Geneva, NY 14456, USA; GanYuan.Zhong@ars.usda.gov

**Keywords:** chilling requirement, dormancy release, FLOWERING LOCUS C, flowering time, MADS-box gene, woody plants

## Abstract

MADS-box transcription factors *FLOWERING LOCUS C* (*FLC*) and *APETALA1* (*AP1*)/*CAULIFLOWER* (*CAL*) have an opposite effect in vernalization-regulated flowering in *Arabidopsis*. In woody plants, a functional *FLC-like* gene has not been verified through reverse genetics. To reveal chilling-regulated flowering mechanisms in woody fruit crops, we conducted phylogenetic analysis of the annotated *FLC-like* proteins of apple and found that these proteins are grouped more closely to *Arabidopsis AP1* than the *FLC* group. An *FLC3-like* MADS-box gene from columnar apple trees (*Malus domestica*) (*MdFLC3-like*) was cloned for functional analysis through a constitutive transgenic expression. The *MdFLC3-like* shows 88% identity to pear’s *FLC-like* genes and 82% identity to blueberry’s *CAL1* gene (*VcCAL1*). When constitutively expressed in a highbush blueberry (*Vaccinium corymbosum* L.) cultivar ‘Legacy’, the *MdFLC3-like* induced expressions of orthologues of three MADS-box genes, including *APETALA1*, *SUPPRESSOR OF OVEREXPRESSION OF CONSTANS 1*, and *CAL1*. As a consequence, in contrast to the anticipated late flowering associated with an overexpressed *FLC-like*, the *MdFLC3-like* promoted flowering of transgenic blueberry plants under nonchilling conditions where nontransgenic ‘Legacy’ plants could not flower. Thus, the constitutively expressed *MdFLC3-like* in transgenic blueberries functioned likely as a blueberry’s *VcCAL1*. The results are anticipated to facilitate future studies for revealing chilling-mediated flowering mechanisms in woody plants.

## 1. Introduction

Exposure to low temperatures (i.e., vernalization) promotes the transition from vegetative to reproductive phase of plant development in most annually flowering species [1,2,3,4]. MADS-box transcription factors *FLOWERING LOCUS C* (*FLC*) and *APETALA1* (*AP1*)/*CAULIFLOWER* (*CAL*) perform an opposite function in vernalization-regulated flowering initiation in *Arabidopsis* [5,6]. *FLC* represses the expression of two major flowering genes, *FLOWERING LOCUS T* (*FT*) and *SUPPRESSOR OF OVEREXPRESSION OF CONSTANS 1* (*SOC1*) [5,6,7,8]. Constitutive expression of *FLC* often delays flowering and causes abnormal floral morphological changes, such as short stamens, reduction of pollen amount, and larger carpels in transgenic plants [6,9,10,11,12]. In contrast, *AP1/CAL* positively promotes the expression of floral meristem identity genes in synergy with *FRUITFULL* (*FUL*) and *LEAFY* (*LFY*) [13,14]. *LFY* was shown to directly activate the expression of *AP1/CAL* in floral primordial and then produce a positive feedback effect on its own expression [13]. Overexpression of *AP1* gene promoted transition from apical or lateral shoots into flowers and early flowering phenotype in transgenic *Arabidopsis* [15]. Genetic evidence showed that either single or double *AP1/CAL* mutants led to abnormal floral morphology in flower development [5,16]. Ectopic expression *AP1* genes in other plant species also showed early flowering phenotypes. For example, constitutive overexpression of *AtAP1* in citrus showed flowering initiation in the first year [17]. Overexpression of apple *AP1-like* genes, *MdMADS2* and *MdMADS5*, induced early flowering in both tobacco and *Arabidopsis* [18]. Overexpression of birch *BpAP1* in tobacco and birch also generated early flowering and dwarf phenotypes [19].

Chilling requirement refers to fulfilling a minimum period of chill hours to stimulate dormancy release and ensure seasonal growth of vegetative and floral buds in a fruit-bearing tree. The chilling requirement for dormancy-break is species- and genotype-dependent [20,21]. Insufficient chilling prevents bud-break and often leads to reduced fruit production mainly due to abortive flower buds and prolonged bloom. To date, unlike the extensively studied vernalization pathway in annual plants, the chilling-involved flowering mechanism in woody plants is largely not known [22,23,24].

Climate changes in the last 40 years have caused earlier shifts in the onset of growing seasons and increased temperature fluctuations [25]. Consequently, early onset of the growing seasons causes insufficient chilling for fruit trees, and increased temperature fluctuations during plant bloom turn early season frosts into freezing injuries to flowers and young fruits [26]. To secure deciduous fruit production, manipulating chilling requirements and cold/freezing tolerance through plant breeding is considered to be a long-term solution to mitigate the potential threats of climate change [27]. Developing cultivars with a low chilling requirement through modifying the flowering initiation pathway may expand the cultivation areas of temperate deciduous fruit trees in warm areas and may also secure high profitability of protected cultivation in greenhouses [28,29,30].

Apples (*Malus domestica*) and highbush blueberries (*Vaccinium corymbosum* L.) need sufficient chilling exposure to release bud dormancy. Modifying the flowering initiation pathway is one of the most important improvement properties for these two fruit crops. In apples, transcription profiling of chilled buds has revealed the potential roles of *FLC-like*, *FT-like* and *TERMINAL FLOWER 1-like* (*TFL1-like*) genes [31,32]. To date, functional *FLC* orthologues have not been identified in woody fruit crops. To study the molecular mechanism of chilling-mediated flowering in woody plants, we cloned an *FLC-like* MADS-box gene from columnar apple trees (*MdFLC3-like*). Functional analysis of the *MdFLC3-like* through constitutive expression was conducted in transgenic highbush blueberry cv. Legacy. Surprisingly, we found for the first time that constitutive expression of the *MdFLC3-like* in transgenic blueberry plants promoted flowering under nonchilling conditions where nontransgenic could not flower. The results are anticipated to facilitate future studies for revealing chilling-mediated flowering mechanisms in woody plants.

## 2. Results

### 2.1. Isolation and Sequence Analysis of an MdFLC3-like Gene

**Figure 1:**
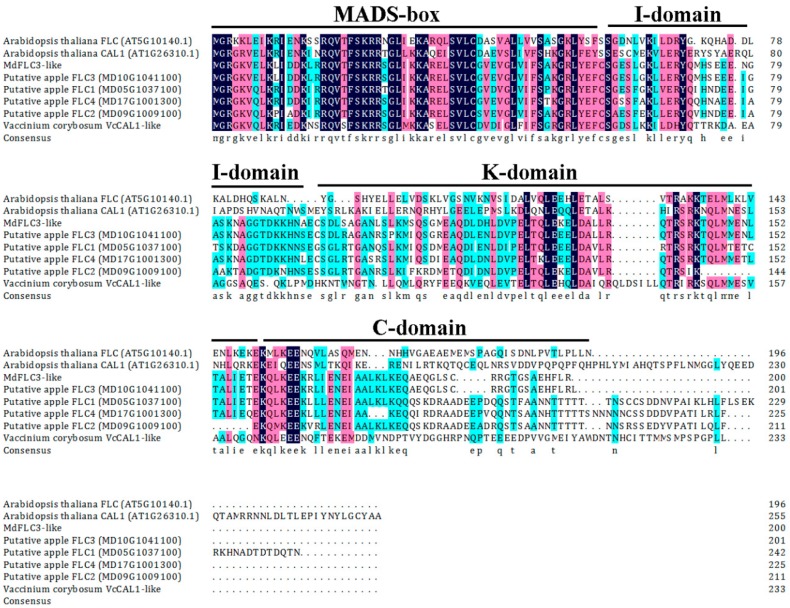
Alignment of *Malus domestica FLOWERING LOCUS C (MdFLC3-like)* (apple *FLC-like*) and its orthologues in apple, blueberry, and *Arabidopsis*.

**Figure 2:**
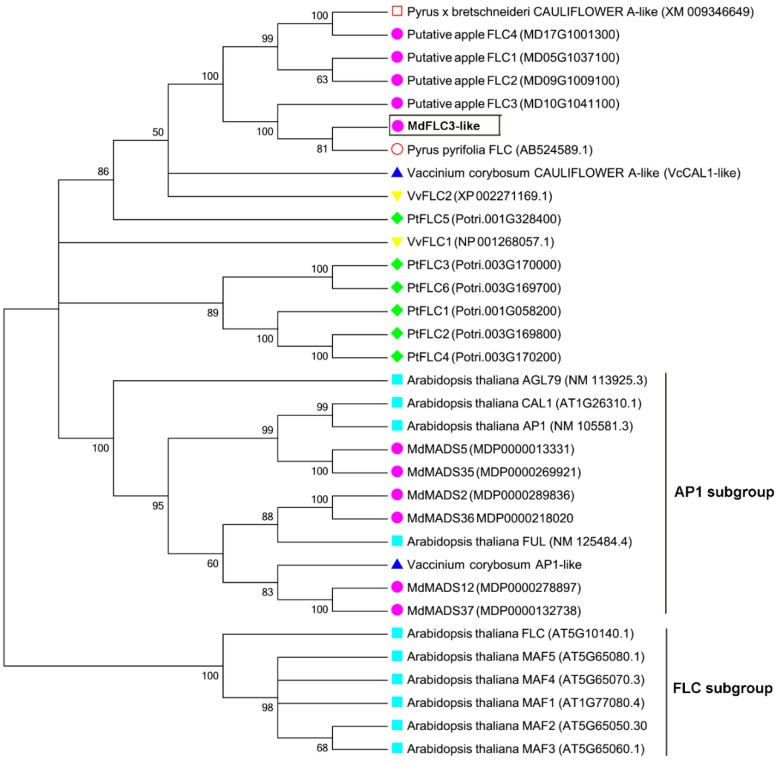
Phylogenetic analysis of *FLC-like* proteins of woody plants and *Arabidopsis FLC* and *APETALA1 (AP1)* proteins. A phylogenetic tree of these MADS-box genes was generated by the neighbor-joining (NJ) method with 1000 bootstrap replicates. The proteins were clustered and divided into two distinct clades, *FLC* and *AP1*.

In the previous study of early flowering in column apple trees, a putative *FLC-like* candidate gene was identified from apples using gene-specific primers based on the apple *FLC3* sequence MD10G1041100 retrieved from *Malus × domestica* GDR RefTrans V1 [33]. The low expression of the *FLC-like* seemed to be responsible for the early flowering and bud dormancy release [31,33]. The cloned *FLC-like* cDNA sequence codes 200 amino acid residues (GenBank accession number: MK986790); its molecular mass is projected to be 22.42 kDa and isoelectric point is 8.15. It showed 95% identity to MD10G1041100 at protein level. An NCBI Conserved Domain Database search revealed that this *FLC-like* cDNA possesses several typical conserved domains including MADS-box, I-domain, K-domain and C-domain, which is a MIKC^c^-type MADS-box transcription factor (Figure 1). In a phylogenetical analysis, the protein of this cDNA was grouped into the *FLC* clade along with *FLC-like* proteins of *Arabidopsis*, grape, pear, and poplar (Figure 2); for example, it shows a high identity to a Japanese pear (*Pyrus pytifolia* ‘Culta’) protein annotated as *FLC* (up to 92%). Accordingly, the cloned cDNA was designated as the *MdFLC3-like*. It is interesting that not every protein sequence showing high identity to the *MdFLC3-like* in the phylogenetical analysis was annotated as *FLC-like* (Figure 2). One *MdFLC3-like* orthologue in a Chinese white pear (*Pyrus bretshneideri*) shows 88% identity and was annotated as *CAULIFLOWER (CAL)-like*. Similarly, the *MdFLC3-like* shows 82% identity to a blueberry *CAL (VcCAL1)* in a highbush blueberry (Figure 2). More interestingly, all the annotated *FLC-like* proteins that showed high similarities to the *MdFLC3-like* are grouped more closely to *Arabidopsis AP1* than *FLC* clade (Figure 2). Apparently, the *MdFLC3-like* and its orthologues in other woody plants are all annotated based on identities of their protein sequences while their functions have not been verified through functional analysis.

### 2.2. Expression Levels of MdFLC3-like in Four Apple Tissues

**Figure 3:**
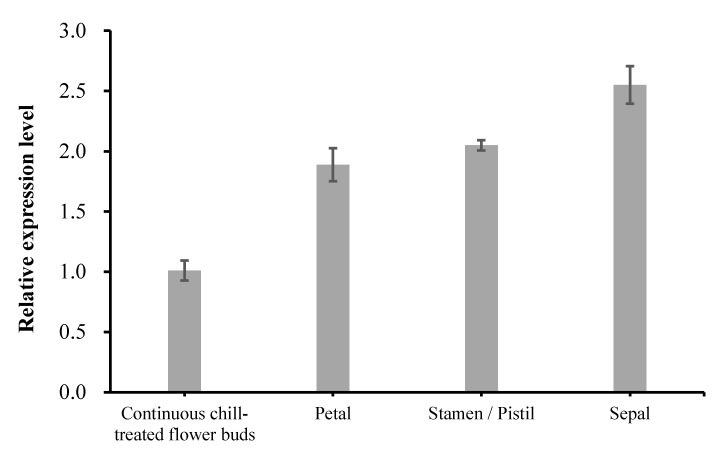
Relative expression levels of *MdFLC3-like* gene in different apple tissues. β-actin gene (GQ339778.1) was used as an internal control for normalization of the transcript levels. Values of gene relative expression levels are means ± SD (standard deviation) of three biological replicates from different trees.

RT-qPCR analysis was conducted to characterize the expression of the *MdFLC3-like* in fully chilled flower buds, petals, stamens/pistils, and sepals. The fully chilled flower buds showed a significant lower *MdFLC3-like* expression than the other three flower tissues (Figure 3). Unlike in *Arabidopsis*, where the expression of *FLC* was barely detected in young tissues of the inflorescence [34], high expressions of the *MdFLC3-like* were observed in apple petals, stamens/pistils and sepals (Figure 3), suggesting that it is not likely that the *MdFLC3-like* functions as an *FLC* function in apples despite being suggested so from its annotation and reduced expression in responding to chill accumulation [35].

### 2.3. Ectopic Expression of MdFLC3-like Promoted Flowering of Nonchilled Blueberry

**Figure 4:**
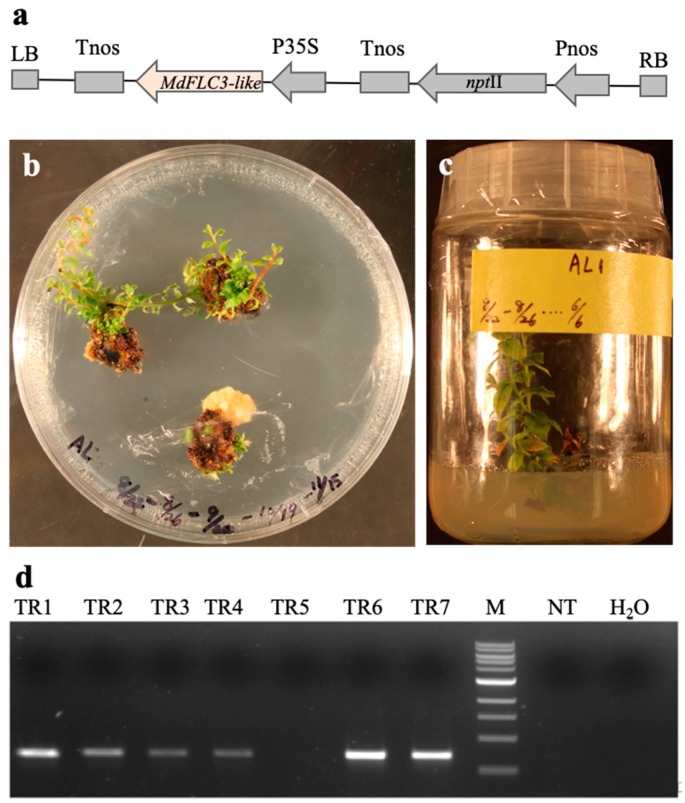
Transformation and ectopic expression of the *MdFLC3-like* gene in transgenic blueberries. (**a**) T-DNA regions of the construct 35S-*MdFLC3-like*. LB: T-DNA left border. RB: T-DNA right border. Tnos: nos terminator. Pnos: nos promoter. P35S: cauliflower mosaic promoter. (**b**,**c**) Selection and regeneration of Km-resistant shoots. (**d**) Genomic PCR detection of *35S-MdFLC* in Km-resistant shoots. TR1–TR7: independent transgenic events. M: 1 kb size marker. NT: nontransgenic shoots. H_2_O: water control.

The *MdFLC3-like* was cloned into the recombinant plasmid pBI121 to make a 35S-*MdFLC3-like* construct of constitutive expression (Figure 4a). The 35S-*MdFLC3-like* construct was transformed into blueberry cultivar ‘Legacy’. A total of 7 out of 232 leaf explants produced Km-resistant shoots, of which six PCR-positive transgenic events were identified (Figure 4b–d).

**Figure 5:**
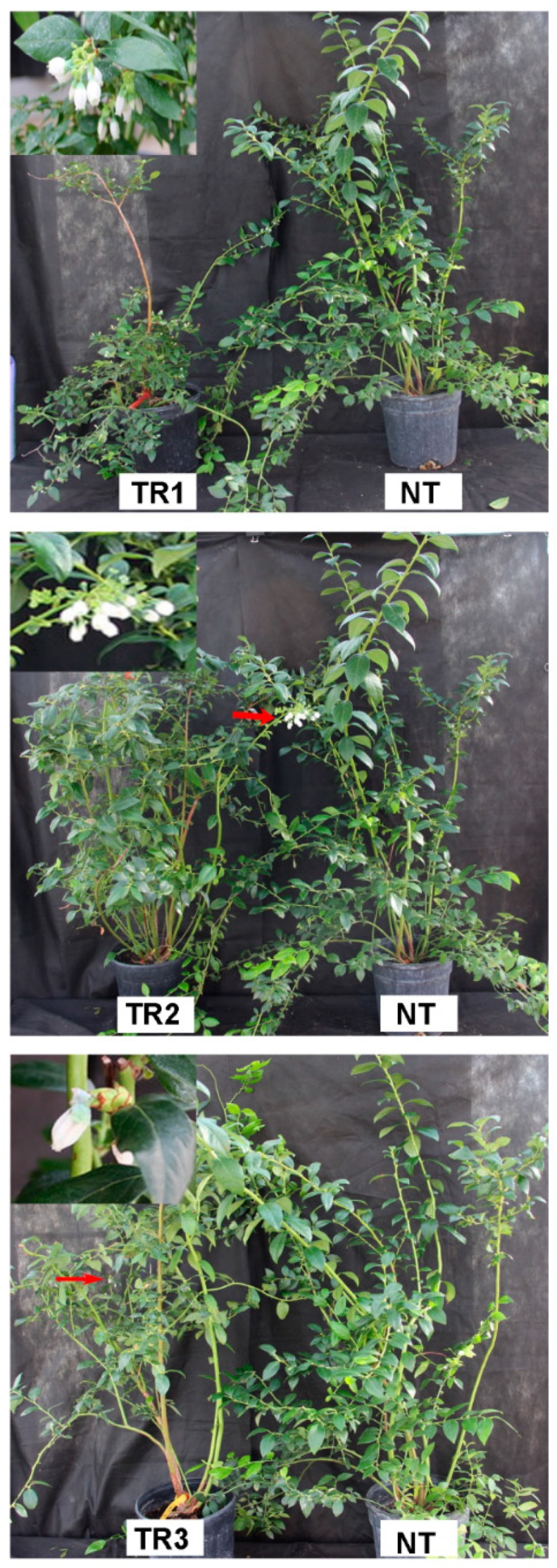
Early flowering of the transgenic blueberry plants carrying 35S-*MdFLC* under nonchilling conditions where nontransgenic (NT) plants could not flower. (**a**–**c**) Early flowering of nonchilled plants of three transgenic events (TR1, TR2, and TR3) in the third year after being transferred to soil. (**d**) NT plant (right) that could not flower despite the appearance of flower buds.

In vitro-rooted plants of three randomly selected transgenic lines (TR1, TR2 and TR3) and three nontransgenic (NT) ‘Legacy’ plants were morphologically normal and they were planted for phenotyping analysis. For one-year-old plants grown in the greenhouse, the TR1 plant showed dwarfing, whereas both the TR2 and TR3 plants were morphologically similar to the NT plants. For the two-year-old plants, fully chilled plants of all transgenic flowered and none of the NT plants had flowers in the spring of 2017, suggesting that the transformed 35S-*MdFLC3-like* promoted flowering. More surprisingly, some newly formed flower buds of transgenic plants (3-year-old) flowered in October of 2017 in the greenhouse prior to their chill accumulation (Figure 5); in contrast, none of the 3-year-old NT ‘Legacy’ plants showed flowers. The flowers were normal in morphology for all three events. The dwarfing transgenic event TR1 developed more flower clusters than the other two transgenic lines (Table 1), and the flowers were eventually developed into fruits (Figure 5). TR2 and TR3 produced fewer flower clusters (Table 1). Overall, the transgenic plants had more flower buds than the NT plants (Table 1). Apparently, constitutive expression of the 35S-*MdFLC3-like* functioned as a flowering promoter under nonchilling conditions; the results are in contrast to those of *Arabidopsis* in which an overexpressed *FLC* delays flowering [6].

### 2.4. Responses of Flowering Pathway Genes to the Constitutive Expression of *MdFLC3-like*

**Figure 6:**
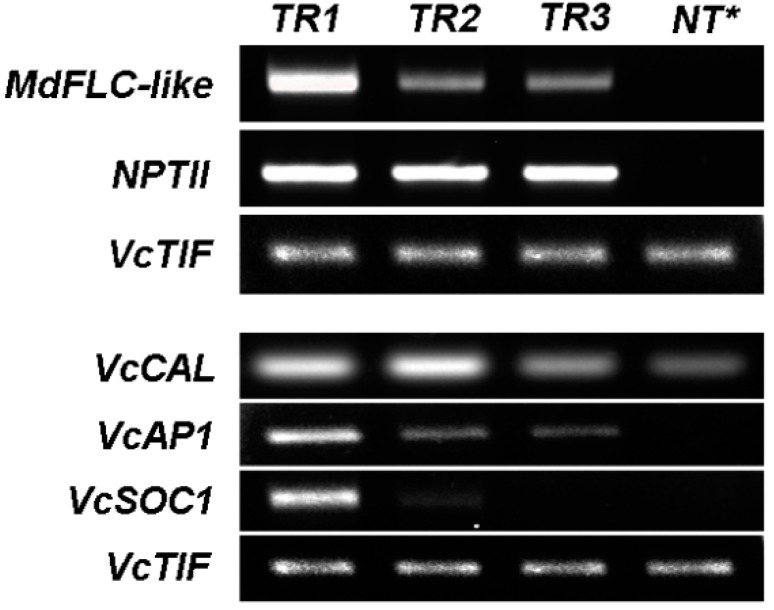
Expression analysis of 35S-*MdFLC* and several selected flowering pathway genes in the fully-expanded leaf tissues of transgenic blueberry plants. TR1, TR2, and TR3: three independent transgenic events. NT: nontransgenic plants, *cDNA mixture of three NT plants. *VcTIF* was used for normalization of the transcript levels. All samples were technically repeated at least three times.

Constitutive expression of the *MdFLC3-like* was confirmed in all three transgenic events but was absent in NT plants (Figure 6). The dwarfing TR1 plants showed a higher expression of the *MdFLC3-like* gene than TR2 and TR3, suggesting that the higher expression was responsible for the dwarf plants with the most promoted flowering (Table 1).

Due mainly to the lack of real biological replicates for each of the three transgenic lines at the time of analysis, the transcript levels of *VcCAL1* and its major downstream genes in newly-formed, fully-expanded leaves were quantified using semi-quantitative RT-PCR. Expression of *VcAP1* was detected in all three transgenic plants but absent in the NT plants, suggesting that the increased *VcAP1* could be the major cause of the promoted flowering in the three transgenic plants. Increased *VcCAL1* and *VcSOC1* (compared to the NT plant) were detected in TR1 and TR2 but not in TR3 plants. Expression of *VcLFY* was hardly detectable in either transgenic or NT plants. The TR1 showed higher expressions of both *VcAP1* and *VcSOC1* than TR2 and TR3, which might contribute to the more significant phenotypic changes in plant size and flowering time in the TR1. Apparently, constitutively expressed *MdFLC3-like* acted as a positive regulator of flowering by enhancing expression of *VcAP1 or VcSOC1*.

## 3. Discussion

### 3.1. A Functional FLC Orthologue in Woody Plants is Unknown

*FLC* functions as a floral repressor during flowering initiation and it is down-regulated after vernalization [1,3,6,7,8,11]. *FLC-like* genes are present in a multi-gene subfamily in most of the annual plants and have been identified in several woody plants based on sequence similarities, including four in poplar (*PtFLC2–5*) [36,37], two in grapevine (*VvFLC1* and *VvFLC2*) [38] and one in peach (*Ppe MADS08*) [39]. Using the *Arabidopsis FLC* as a query, orthologues of the *FLC* have not been identified in apple, apricot [40], cucumber [41], or blueberry [42]. However, when the phylogenetic analysis was conducted with the orthologs of poplar, peach, and grapevine MADS-box genes, four apple genes (i.e., *MD09G1009100*, *MD17G1001300*, *MD05G1037100*, and *MD10G1041100*) were clustered in the same group together with poplar, peach, and grapevine *FLC-like* genes [35,43]. To date, none of these *FLC-like* genes have been verified through a functional analysis. Thus, no convincing evidence has shown that a functional *FLC-like* gene plays a major role in chilling-mediated flowering in woody plants.

### 3.2. MdFLC3-like Does Not Function as an FLC in Blueberry

The *MdFLC3-like* isolated from apples shows a high identity to *MD10G1041100*, which was annotated as an *FLC-like* gene in apples based on sequence analysis and characterization of its expression in apples [36]. Since the expression of *Arabidopsis FLC* was hardly detected in the young tissues of inflorescence [35], a functional *FLC-like* is anticipated to have a low or no expression in inflorescence. However, the high expression levels of *MdFLC3-like* in different apple flower tissue (i.e., sepals, petals, stamen, and pistil) suggest that the *MdFLC3-like* in apples is likely different from the *FLC* in *Arabidopsis*. Additionally, despite of being grouped in the *FLC-like* clades (Figure 2), not all of the *FLC-like* orthologues identified in woody plants showed the similar transcriptional expression pattern as *FLC*. For example, *PtFLC2* and *PtFLC4* in poplar showed opposite expression patterns during bud dormancy release [37,39]. The *PtFLC2* expression decreased in response to chilling during winter dormancy and increased after dormancy release [37]. A similar expression pattern was observed for grapevine *VvFLC1* [39]. Our work showed that the *MdFLC3-like* expression was similar to that of *PtFLC2* and *VvFLC1* [36]. For the poplar *PtFLC4* and grape *VvFLC2*, their expressions were increased during dormancy and decreased after cold exposure [37,39].

*FLC* is a repressor of flowering through repressing its downstream *SOC1*, *SPL15*, and *FT* by directly binding to the promoters of *SOC1* and *SPL15* or the first intron of *FT* [44,45]. Overexpression of *FLC* resulted in a late-flowering phenotype in *Arabidopsis* and *Brassica rapa* [9]. Silencing *BcFLC2*, the homolog of *FLC* in Pak-choi (*Brassica rapa* ssp. Chinensis) caused early flowering [46]. In this study, the fact that constitutively expressed *MdFLC3-like* promoted blueberry flowering under nonchilling conditions supports that *MdFLC3-like* has little *FLC* functions as a repressor in chilling-mediated flowering. The observed phenotypic changes seem to be well supported by the expression levels of the *MdFLC3-like* and the enhanced expression of *VcAP1* and *VcSOC1*.

### 3.3. MdFLC3-like Likely Functions in a Positive Role in Flowering Initiation in Transgenic Blueberry

Based on transcriptome reference (GenBank accession number: SRX2728597) of blueberry cultivar ‘Legacy’, several MADS-box genes were found to show similarity to the *FLC*; however, none of them were annotated as an *FLC-like* due to their higher similarities to the other MADS-box genes [24,42]. For example, using the *MdFLC3-like* as a query to search the blueberry transcriptome reference, the best hit was actually the *VcCAL1* (up to 82% identity). Similarly, the *MdFLC3-like* orthologue in a Chinese white pear was also annotated as a *CAL-like* (Figure 2).

In nonchilled tissues of blueberry ‘Legacy’, the expression of *VcCAL1* was the highest in flower tissues, followed by leaves and buds [42]. This is consistent with the tissue specificity and developmental stage of the *CAL* expression in *Arabidopsis* [5,13]. In addition, from nonchilled to fully chilled flower buds, a significant increase in *VcCAL1* expression occurred, and then a decrease in the expression was found in late-pink buds [24]. Apparently, the expression pattern of the *VcCAL1* in blueberries is similar to that of the *CAL* in *Arabidopsis* but is different from that of either the *MdFLC* expression in apples or *FLC* expression in *Arabidopsis* [1,3,6,7,8,11,35].

*CAL* is considered functionally to be partially redundant to *AP1* and acts as a positive regulator of flower development in *Arabidopsis* [5,13]. Constitutive expression of the *MdFLC3-like* promoted flowering in transgenic blueberries, suggesting that the *MdFLC3-like* does not function as an *FLC* in blueberries as expected. This was further supported at transcript levels, where the upregulated expression of *VcAP1* and *VcSOC1* was observed only in the transgenic plants (Figure 5 and Figure 6).

### 3.4. MADS-Box Genes Play Key Roles in Chilling-Mediated Flowering of Woody Plants

*FLC* is a MADS-box gene. Another MADS-box gene cluster named *DORMANCY-ASSOCIATED MADS-box* (*DAM*) genes in peaches were proposed as candidates for regulation of the terminal bud’s formation in response to dormancy-inducing conditions [47]. However, the *DAM* genes are the orthologues of *A. thaliana AGL24* and *SVP* genes [48,49] rather than the orthologues of *FLC*. More recently, the *SHORT VEGETATIVE PHASE-LIKE* (*SVP-like*) of hybrid aspen, an orthologue of *A. thaliana SVP*, was reported to be involved in axillary bud dormancy as a mediator of temperature controlled bud break [50]. Hence, it is likely that multiple MADS-box genes, instead of any individual *FLC-like* or *DAM* genes, confunction to determine the process of chilling-mediated flowering in woody plants. In blueberries, the changes from nonchilled to chilled and chilled to late-pink buds are associated with transcriptional changes in a large number of differentially expressed (DE) flowering pathway genes (i.e., the orthologues of *FT*, *FD*, *TFL1*, *LFY*, and other MADS-box genes) [24]. In general, fully chilling upregulates blueberry MADS-box genes [24]. It is interesting that the functional orthologues of *FLC* and *AGL24* were not detected in blueberries [24]. In addition, overexpression of the keratin-like (K) domain of the *VcSOC1* promoted flowering of the blueberry cultivar ‘Aurora’ under nonchilling conditions [51]. It is likely that the interaction of multiple MADS-box genes, rather than a single *FLC-like* gene, co-regulates chilling-mediated flowering in blueberries as well as in other woody plants. Further studies are still needed to reveal roles of MADS-box genes in chilling-mediated flowering of woody plants.

## 4. Material and Methods

### 4.1. Plant Material

Six-year-old columnar apple trees (*Malus domestica*) from a cross of ‘Gala’ × ‘Telamon’ were used for gene cloning and expression analysis in this study. These trees were grown at the Laiyang Experiment Station of Qingdao Agricultural University in Shandong Province, China. Chilled flower buds, 40–50 per tree, were collected in March 2011. Flower tissues (e.g., sepals, petals, stamens, and pistils), about 200 mg per tree, were harvested in early May 2011. Samples of three biological controls from three trees were collected and immediately frozen in liquid nitrogen and stored at −80 °C for later RNA extraction.

In vitro ‘Legacy’ shoots were cultured on 30 mL WPM2Z in 40 mm × 110 mm glass jars and incubated for 4 weeks at 25 °C, 30 µE/m^2^/s of 16 h/8 h (day/night); rooting of in vitro cultured shoots and greenhouse care of rooted plants were conducted according to the published protocols [52]. All plants were grown in a greenhouse (heated for winter) under natural light conditions with routine management of watering with added 0.2 g/L fertilizer (nitrogen:phosphorus:potassium = 21:7:7) unless otherwise mentioned. All blueberry experiments were conducted at Michigan State University (East Lansing, Michigan, USA).

### 4.2. MdFLC3-like Cloning and Phylogenetic Analysis

Total RNA was isolated from nonchilled apple flower buds using the RNeasy Plant Mini Kit (Qiagen, Valencia, CA, USA) [33]. Total RNA (0.5 μg) was reverse transcribed into complementary DNA (cDNA) using SuperScript II Reverse Transcriptase (Invitrogen, Carlsbad, CA, USA). The open reading frame (ORF) of the *MdFLC3-like* gene was cloned by PCR amplification reaction using GoTaq Green Master Mix (Promega, Madison, WI, USA). Gene-specific primers on the basis of the sequence MD10G1041100 were retrieved from *Malus × domestica* GDR RefTrans V1 and used to amplify *MdFLC3-like* (Table 2). The PCR was carried out with the following protocol: an initial denaturation of 94 °C for 5 min; 35 cycles of 94 °C for 30 s, 58 °C for 30 s, and 72 °C for 1 min; and a final extension of 72 °C for 7 min. The deduced MdFLC protein sequence was characterized using CLC sequence viewer 7 (QIAGEN Bioinformatics, Aarhus, Denmark).

Amino acid sequences of *MdFLC* orthologs were retrieved using the NCBI server (http://blast.ncbi.nlm.nih.gov/Blast.cgi). In addition, the *MdFLC* was also used as the query to search the transcriptome reference developed for ‘Legacy’ using Trinity and Trinotate (GenBank accession number: SRX2728597) [42]. Selected orthologs were aligned using the ClustalX (http://www.clustal.org). A phylogenetic tree was generated using the MEGA5 [53].

### 4.3. Quantitative Reverse Transcription PCR (RT-qPCR) Analysis

To determine the expression levels of *MdFLC3-like* in different apple tissues, total RNA was isolated from nonchilled and chilled buds, sepals, petals, stamens, and pistils. For RT-qPCR analysis, 1 μg aliquot of total RNA treated with DNase I (Invitrogen) was reverse-transcribed using SuperScript II Reverse Transcriptase (Invitrogen). Real-time PCR using SYBR Green PCR Core Reagents (Life Technologies, Carlsbad, CA, USA) was carried out on an Applied Biosystems StepOne™ thermocycler (Applied Biosystems, Foster City, CA, USA) with three technical replicates per sample. The amplification conditions were 95 °C for 10 min, followed by 40 cycles of amplification (95 °C for 15 s, 60 °C for 1 min) with plate readings after each cycle and performance of a melting curve analysis. Primers used for the reactions are provided in Table 2. Preliminary experiments were conducted to determine the efficiencies of the primers and confirm that the internal control was suitable. Data was analyzed by the 2^−ΔΔ*Ct*^ method using StepOne Software V2.2 (Applied Biosystems).

### 4.4. Construction of a MdFLC3-like Expression Vector

To make a construct for constitutive expression, the *MdFLC3-like* fragments (5′-*BamH*I–*MdFLC3-like*–*EcoR*V-3′) containing two added restriction enzyme sites were amplified by specific primers MdFLC-BF and MdFLC-ER (Table 2) from the apple cDNA. The purified PCR fragments were released by double digestion, and the digested 5′-*BamH*I–*MdFLC3-like*–*EcoR*V-3′ fragments were purified. Plasmid pBI121 was digested with *Sac*I; the *Sac*I-digested fragments were incubated by DNA Polymerase I Large Klenow Fragment (NEB, Ipswich, MA, USA) to generate blunt-*Sac*I ends. The digested pBI121 containing blunt-*Sac*I ends were digested with *BamH*I to remove the *gus*A coding region. The opened *Bam*HI and blunt-*Sac*I sites in the T-DNA region between the *cauliflower mosaic virus* (CaMV) 35S promoter and the Nos terminator in pBI121 allows the insertion of the digested 5′-*BamH*I–*MdFLC3-like*–*EcoR*V-3′ fragments (Figure 4A). The resulting 35S-*MdFLC3-like* was sequenced and introduced into *Agrobacterium tumefaciens* stain EHA105 [54] using the freeze-thaw method.

### 4.5. Transformation of 35S-MdFLC3-like to ‘Legacy’

Transformation of ‘Legacy’ was performed as previously reported [52]. To identify transgenic blueberry events, DNA and RNA were extracted from leaf tissues according to protocols reported previously [55]. Two pairs of primers, NPTII-F and NPTII-R as well as 35S-F and MdFLC-ER (Table 2), were used to detect the presence of *npt*II and *MdFLC3-like* genes separately.

Since the tetraploid southern highbush blueberry ‘Legacy’ needs over 800 chilling units (CU) for normal flowering, it will not flower under the above greenhouse conditions without a chilling treatment. The total CU was calculated according to Norvell et al., 1982 [56]. Both transgenic and nontransgenic ‘Legacy’ shoots were directly rooted in 48-cell trays containing sphagnum peat moss [52]. Due to the concerns of their vulnerability to freezing, these rooted one-year-old plants were repotted into 2-gallon pots in October of 2015 and grown in a greenhouse with heating under natural light conditions during the winter of 2015–2016. After the winter, the plants were moved to a secured courtyard and the plants were grown under natural conditions through the winter of 2016–2017. Then prior to any chilling accumulation in August of 2017, the three-year-old plants were moved back again to the greenhouse during the winter of 2017–2018; meanwhile, six six-year-old nontransgenic ‘Legacy’ with visible flower buds in them were also moved to the greenhouse. Plant growth and flowering time were documented.

### 4.6. Gene Expression Analysis of Transgenic ‘Legacy’

To compare the expression levels of *MdFLC3-like* and other flowering genes in transgenic and nontransgenic blueberries, three transgenic events and wild-type blueberries were studied. Fully-expanded leaves near the shoot tips, about one gram from each plant, were randomly collected from TR1, TR2, TR3, and three nontransgenic plants (NT). The leaf samples were grounded in liquid nitrogen and 200 mg per sample were used for total RNA isolation. A two-step semi-quantitative RT-PCR was performed using SuperScript II Reverse Transcriptase (Invitrogen) for reverse transcription and GoTaq Green Master Mix (Promega) for PCR amplification. For the NT plants, cDNA mixtures from three plants were used in semi-quantitative RT-PCR analysis. Since there was only one plant for each transgenic event in this investigation, RT-qPCR was not conducted due to the lack of biological replicates. The sequences of the specific primers to distinguish the expression of *MdFLC3-like*, *VcCAL1*, *VcAP1*, *VcSOC1*, and *VcLFY* were listed in in Table 2. *VcTIF* was used as an internal control and normalizing reference for each gene in all samples. The PCR reactions were performed in triplicate and the PCR-amplified gene products were detected in a 2% agarose gel.

## 5. Conclusions

As an initial step to investigate the potential roles of an *FLC-like* gene in woody plants, we cloned an apple *FLC-like* gene (*MdFLC3-like*), which shows a high similarity to blueberry’s *VcCAL1*. Ectopic expression of the *MdFLC3-like* likely promoted flowering of nonchilled transgenic plants by enhancing expression of *VcAP1* and *VcSOC1*, both of which were upregulated in the chilled flower buds of nontransgenic plants [24]. These results suggest that the *MdFLC3-like* gene functioned as *VcCAL1*, a positive regulator of flowering in transgenic blueberries.

## Figures and Tables

**Table 1:** Total number of flower buds, number of flower clusters, and average flower number for each cluster in transgenic and nontransgenic (NT) plants in November of 2017 in the greenhouse prior to their chill accumulation. * Average of three plants.

	Total Number of Flower Buds	Number of Flower Clusters	Average Flower Numbers/Cluster
TR1	52	37	4.7
TR2	23	3	7.6
TR3	26	1	2
NT *	16.3	0	0

**Table 2:** Primers used in this study.

Primer Name	Primer Sequence (5′ to 3′)	Products Size
**Primers for Gene Cloning**		
MdFLC-F	ATGGGGCGAGGGAAGGTAGAGC	603 bp
MdFLC-R	TTATCGGAGGAAGTGCTCTGCT	
MdFLC-BF	CGGATCCATGGGGCGAGGGAAGGTAGAGC	614 bp
MdFLC-ER	CGATATCTTATCGGAGGAAGTGCTCTGCT	
**Primers for RT-qPCR and Semi-Quantitative RT-PCR**		
MdFLC-Fr	ATAATGCGGAATGTAGTG	151 bp
MdFLC-Rr	CTTGTTTGTCTTAGAAGTG	
VcAP1-Fr	AAGGAACATAAGGCACTAT	145 bp
VcAP1-Rr	AAGGTCAGAGATAGATTCAT	
VcLFY-Fr	CTGGACGATATGATGAAC	166 bp
VcLFY-Rr	GAGCATGTGTAGGAGTAT	
VcSOC-Fr	CCAAGAGGAAAGCTCTACGA	550 bp
VcSOC-Rr	ATTGCACGTATCCAATGCTT	
VcCAL1-Fr	AATGGCACTAACCTACTC	112 bp
VcCAL1-Rr	GTTGTATGGCATCTAGTTG	
VcTIF-F (Eukaryotic translation initiation factor 3 subunit H)	GAGAGATTCAGATGCCCAGAAG	355 bp
VcTIF-R	GGACAATGGATGGACCAGATT	
**Primers for Transgenic Plants Detection**		
NPTII-F	GAGGCTATTCGGCTATGACTG	701 bp
NPTII-R	ATCGGGAGCGGCGATACCGTA	
35s-F	TGACGCACAATCCCACTATC	714 bp
MdFLC-R	TTATCGGAGGAAGTGCTCTGCT	
